# Investigation on the Cooling and Evaporation Behavior of Semi-Flexible Water Retaining Pavement based on Laboratory Test and Thermal-Mass Coupling Analysis

**DOI:** 10.3390/ma12162546

**Published:** 2019-08-09

**Authors:** Qiang Dong, Chonghui Wang, Chuhua Xiong, Xiulei Li, Hainian Wang, Tianqing Ling

**Affiliations:** 1 School of Civil Engineering, Chongqing Jiaotong University, Nan’an, Chongqing 400074, China; 2 Institute of Highway Engineering, RWTH Aachen University, Mies-van-der-Rohe-Street 1, 52074 Aachen, Germany; 3 School of Highway, Chang’an University, Xi’an 710064, China; 4 College of Traffic and Transportation, Chongqing Jiaotong University, Nan’an, Chongqing 400074, China; 5 National and Local Joint Engineering Laboratory of Traffic Civil Engineering Materials, Chongqing Jiaotong University, Chongqing 400074, China

**Keywords:** pavement engineering, road surface, semi-flexible water retaining pavement, thermal-mass coupling, numerical simulation, evaporation

## Abstract

The Semi-Flexible Water Retaining Pavement (SFWRP) has the capability to cool down the temperature of the road surface through its evaporation behavior, including absorbing and evaporating water; this is an efficient approach to relieve the heat island effect in a big city. The temperature feedback from different material surface were investigated in this paper in the same test condition, it has been proved that the SFWRP material can remarkably cool down the temperature of the road surface. The mechanism of the material evaporation behavior, including flux calculation formula of the water vapor inside the air void, were studied by inter-phase continuous function, in which the structural properties of the SFWRP material was taken into account. Furthermore, the function calculating the evaporation of the water vapor was then developed in this research through heat and mass transfer analogy. Besides, the calculating results can be captured by the self-coding program in Finite Element Modeling (FEM) for water evaporation simulation. Also, the results of laboratory tests were adopted to validate the calculating model. Finally, it has been proved that the mortar was recommended to be used in semi-flexible water retaining pavement to serve as material with permeable and water retaining property, and the semi-flexible water retaining pavement material is recommended to applied in the surface layer of the permeable pavement.

## 1. Introduction

### 1.1. Background

The Heat Island Effect has become a frequently concerning issue in recent times due to the acceleration of the urbanization and the development of industrial construction. This phenomenon also involves the issues concerning to the pavement performance [1,2,3,4,5]. The Semi-Flexible Water-Retaining Pavement (SFWRP) has been proved as one of the approaches which can effectively deal with this problem. The SFWRP is one sort of composite material which uses asphalt mixture with large porosity (where the air void is from 20% to 25%) as base material, and the water-retaining mortar with fixed content is then infused into the asphalt mixture to finally harden into a composite material. The water-retaining mortar can automatically store water when it rains or when the road is sprinkled. When there is an increase of temperature on the road surface, the water will evaporate and consume the heat of the pavement, thereby reducing the temperature of the road surface and the near-ground atmosphere. The temperature reduction can even be up to 10 °C or more [6]. However, it is necessary to estimate evolution of water evaporation inside SFWRP material, and how does this phenomenon reduce the pavement temperature requires quantitative analysis with its evaporative cooling mechanism.

### 1.2. Mechanism Investigation with Laboratory Test

Recently, the qualitative research about the cooling mechanism of water-retaining pavement has been investigated by several scientists [7]. Sha et al. developed the model in 2012 which was used to characterize the variation in surface temperature of water-retaining specimen according to the regression analysis of the data captured from indoor cooling test, in which the hydrothermal model, which is used for evaluating the evaporation volume of water-retaining pavement under constant temperature and humidity condition, was developed as well [8]. Also, they have calculated the evaporation volume of water-retaining pavement with the aid of semi-infinite simplified model, which indicates the capability of semi-infinite simplified model to assess the moisture evaporation behavior of water retaining pavement. For the comparative analysis of open graded friction course (OGFC) and water-retaining pavement, in 2020, He et al. have investigated the water evaporation volume of specimen and its surface temperature under different condition which is up to the distance from the upper surface of the specimen to the height of sample water-immersed surface; the temperature of sample under complete wetting condition was also taken into account [9]. Also, the calculation model was verified by comparison with the measured value of the test. It was found that the evaporation amount decreased significantly as the water surface distance from the top surface of the test piece increased. Li [10] used the measured evaporation rate to calculate the latent heat flux of the pavement structure. This method can be applied to determine the heat relationship in certain engineering, but due to environmental differences, this method has limitations determined by the environmental condition. In addition, the thermal diffusion equation is directly used to calculate the field temperature of the road surface, namely the water-containing material is regarded as a solid, and the flow and phase change of the fluid inside are not considered, which is inconsistent with the actual situation. Therefore, the existing model or calculation method can only be used with a certain simplified environmental condition, which cannot be applied for general investigation of the water evaporation in SFWRP samples in engineering construction.

### 1.3. Mechanism Investigation with Simulation Investigation

In the field thermal analysis of pavement simulation, Mrawira et al. [11] utilized the analytical element method to derive the temperature response of the layered pavement system under unsteady heat conduction with temperature load. Wang et al. [12] used the separation variable method and Duhamel’s principle to derive the infinite series solution of asphalt pavement temperature field prediction. However, the moisture in the pavement and its latent heat of vaporization and internal convective heat transfer are not considered in these researches, which cannot be applied into the cooling mechanism analysis of SFWRP material.

The evaporation and cooling process of SFWRP material can also be described by the thermal-mass coupling equation in porous media. According to the continuous mass equation in porous media, the coupling equation can be divided into a single-liquid continuous equation model (usually used for soil evaporation), and a continuous equation model for total moisture (usually used for heat and moisture coupling transfer of building walls) and inter-phase continuous equation model (usually used for dry condition). The soil evaporation model [13,14] often uses two independent partial differential equations to control temperature and moisture in the heat and moisture transfer in the soil, the convection and enthalpy migration caused by water flow are considered in this model. In addition, the permeability coefficient of soil is significantly larger than SDWRP material. The thermal-wet coupling model of building walls [15,16,17,18,19] usually combines the liquid water content and the water vapor content into the total moisture content, which is calculated by two control equations of total moisture and temperature coupling. This model does not satisfy the conservation of mass according to literature [20], and it will lead a significant error when the water content increases. It is obvious that the inter-phase continuous equation model is a comprehensive and accurate governing equation. However, after combining the momentum equation and the energy equation, it becomes very complicated and cannot be solved by the analytical method, and the numerical solution cannot be directly used for the solution as well. Therefore, it is necessary to simplify the situation of the continuous equation, and to make it capable for further application of numerical simulation.

From the above, it can be concluded that the existing porous media thermal mass coupling analysis method of permeable pavement cannot be used in the quantitative analysis of evaporating cooling of the SFWRP material.

### 1.4. Approaches and Aims

This essay aims to evaluate the evaporation behavior of SFWRP material by exposure reflection test in laboratory and numerical simulation. A new manufacturing and testing approach of the exposure test was developed in this study. The temperature feedback from different material surfaces were investigated in the exposure test condition. The mechanism of the material evaporation behavior was studied by inter-phase continuous function, in which the structural properties of the semi-flexible was taken into account with the assumption that the volume of the dry air inside the semi-flexible air void keeps stable. The flux calculation formula of the water vapor inside the air void was obtained through inter-phase continuous function. Furthermore, the function calculating the evaporation of the water vapor was then developed in this research through heat and mass transfer analogy, and the calculating results can be captured by the self-coding program in Finite Element Modeling (FEM) software Comsal Multiphics. 

Evaluation models for the moisture evaporation based on a laboratory test and simulation were created and the model parameters were calculated by means of inter-phase continuous function. It can be deduced that the comprehensive conclusions contributed to the increase of accuracy of the prediction of the cooling capacity of SFWRP material, which ultimately can guarantee the sustainable development of cities based on mitigating urban heat island effect.

## 2. Research Methods and Laboratory Test 

### 2.1. Methodology

In this paper, the asphalt mixture and SFWRP samples were manufactured according to Qu and Ling [21,22], and the mix ratio was designed based on the general performance of asphalt mixture and SFWRP samples. The Marshell samples from both mixtures were formed to measure their saturated permeability coefficient, and the rutting plate specimens were formed for the exposure test. The exposure reflection intensity was set based on the field exposure test for the surface layer of asphalt pavement. Then, the same intensity of exposure reflection was used for the SFWRP material, the difference of the evolutions from two samples’ surface temperature were then measured and applied for comparative analysis. 

The mechanism analysis of the material evaporation behavior was investigated based on the inter-phase continuous function, which is simplified according to the actual physical situation. Also, the heat flux and mass flux calculation were taken into account, since the surface of sample is flux boundary. On the other hand, the FEM model was developed considering the initial condition in laboratory and boundary condition, to simulate the evaporating behavior of SEWRP material, and this model was validated by the data captured from exposure test in laboratory. Besides, the model was applied to investigate the variation in water evaporation and surface temperature of SFWRP material under exposure radiation, in which the material with different permeable coefficient was taken into account.

### 2.2. Test Devices and Preparation

The rutting samples of asphalt mixture and semi-flexible water retaining pavement were utilized for the laboratory exposure test, in which the exposure procedure was finished by astigmatic reflector. The reflection intensity was set as the level which can heat the surface of asphalt mixture reached at 60 °C after 2 to 4 h, and this reflection intensity was also used for the exposure of semi-flexible water retaining material. The thermocouple was adopted in this test to measure the temperature from both the top and bottom surface of the sample. For the top side surface, three thermocouples were fixed at the central point and its two sides, respectively, and their spacing is 5 cm. As for the bottom side surface only one thermocouple was fixed at the central point. The thermocouple was fixed with aluminum foil and connected by a flexible connector so that the thermocouple can be disconnected at any time when mass measuring is required. Besides, for the thermal test specimen, the specific heat capacity and thermal conductivity of the material is measured through multifunctional rapid thermal conductivity tester (DRE III, Xiangtan, China); the range of the thermal conductivity measurement is from 0.005 to 100 w/(m·k), and its resolution is 0.005 w/(m·k), while the range of the specific heat capacity measurement is from 0.1 to 5 kJ/(kg·K). The surface heat absorption rate of the test specimen is measured through solar radiation tester, the spectral measuring range of the device is from 300 to 3000 nm, and its sensitivity is 7~14 µV/W·m^−2^. The radiation radiant heat was measured by the net radiation meter with a range of spectral measuring from 0.28 to 50 µm and sensitivity from 7 to 14 µV/W·m^−2^. The moisture characteristic curve of the water retaining semi-flexible material is measured by the 15 bar pressure plate meter.

**Figure 1:**
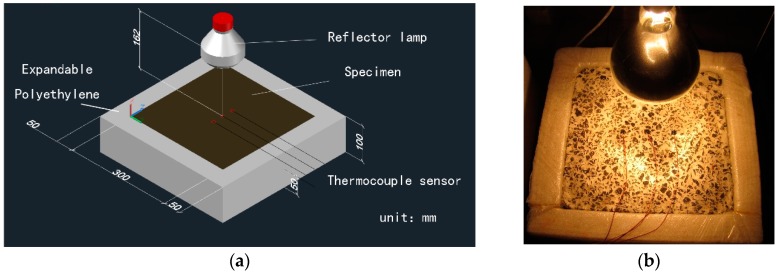
Test sample and device of exposure test: (**a**) Schematic diagram of the exposure test; (**b**) Exposure test in laboratory.

The dried mass of the semi-flexible water retaining material was measured after 48 h of heating in oven with stable condition of 60 °C. As for the humid sample, the mass was measured after its soaking in water for 24 h, and the temperature of the water was stable at 20 °C. The sample stood for 5 min after removing it from water so that there was no more moisture remained on the surface of the sample; the mass of sample was then measured and recorded as its moisture mass. As a consequence, the water absorption per unit volume of the material can be calculated by the comparison between its dried mass and moisture mass. It should be noted that the temperature of the sample surface is measured every 2 min during the exposure test, the mean value of all the thermocouples. Also, the mass of the sample is measured per hour during the test. The test sample and devices of the exposure test in laboratory can be seen in Figure 1.

### 2.3. Laboratory Test

The temperature and relative humidity in laboratory is fixed at 20 °C and 60%, respectively. The reflection intensity and exposure duration of the laboratory test are set according to the real condition of the field pavement with actual exposure. The exposure reflected light source used in this paper is a waterproof reflector with rated power of 200 W. The exposure duration and cooling time are set to be 5 h and 9 h, respectively, which is based on the exposure condition of asphalt mixture in field test. Furthermore, it should be emphasized that the bottom and side of the sample are wrapped by temperature-insulating material, so the heat absorbed from exposure reflection can only be transmitted in the downward direction; this artificial setting is intended to simulate the actual situation of heat transmitting inside pavement, which can only spread downward. On the other hand, the temperature-insulating setting is capable of ensuring the stability and reliability of the measured data due to its thermal insulation performance and its property of preventing the evaporation of water. This setting has access to make full use of the thermal energy, which makes the test more efficient as well as energy saving.

### 2.4. Test Results

**Figure 2:**
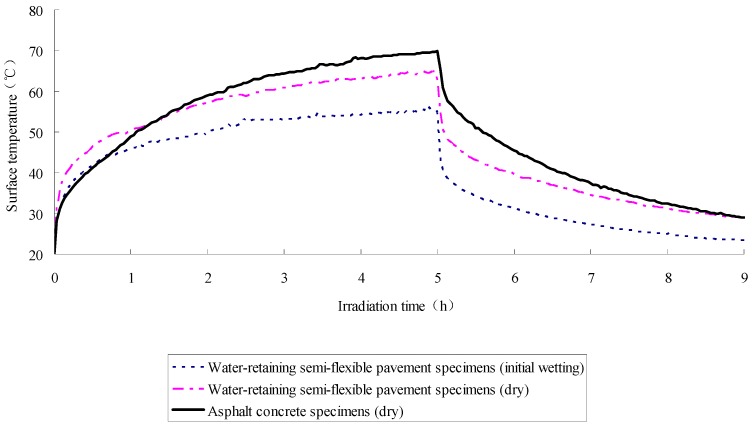
Surface temperature of specimen in exposure test.

**Figure 3:**
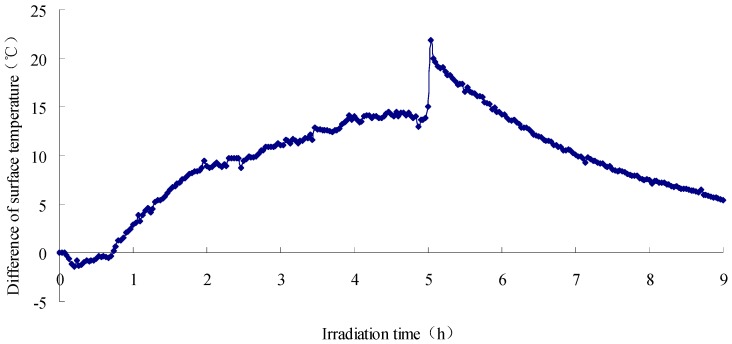
Temperature variation diagram of the Semi-Flexible Water-Retaining Pavement (SFWRP) surface.

The exposure test results of asphalt concrete specimen and water-retaining semi-flexible samples are given in Figure 2, and Figure 3 is the results of calculation. In the figure, the definition of irradiation time is the duration when the specimen was imposed into the exposure reflection test. The exposure reflected light source used here is a waterproof reflector with rated power of 200 W. The exposure duration and cooling time are set to be 5 h and 9 h, respectively, which is based on the exposure condition of asphalt mixture in field test. The ambient temperature in laboratory is 20 °C and the relative humidity is 65%. From this figure, it can be seen that all the specimens yield the same trend curve during the exposure test. Specifically, when the external input heat flux keeps stable, both the heating and cooling process perform that the initial rate of temperature change is fast, and later it becomes slow. Namely, a convex curve is formed at the phase of temperature rise, and a concave curve is formed during the temperature drop.

It can be concluded from Figure 2 and Figure 3 that the variations of sample surface temperature have distinguished features from one another, to be more precise, the SFWRP material has a more efficient function in cooling down the temperature when compared with the asphalt concrete specimen. The maximum decline of SFWRP surface temperature during exposure is 17.7 °C according to the diagram, while the maxi-mum drop of surface temperature during cooling time (when the exposure lamp is shut off) is 21.9 °C. Besides, it can be noted that the SFWRP material performs better than asphalt concrete in cooling even without water retaining, since the heat reflectivity of the SFWRP material is greater than that of asphalt concrete, and the total heat absorbing of SFWRP is less than which of asphalt concrete. Therefore, the SFWRP material can cooling down the surface temperature due to moisture evaporation and low rate of heat absorption.

It also has been found that during the test, there is no significant variation in temperature between SFWRP and asphalt concrete when the surface temperature of specimen is no higher than 40 °C. This is because the water evaporation is not active when the temperature is at a low level. Hence, from this observation, it can be seen that the evaluation of the cooling capability of SFWRP material is not efficient until the surface temperature of the sample is higher than 40 °C; this condition can be satisfied in field test at noon or later during the day time (for an area where the sun exposure temperature is higher than 40 °C during day time).

The SFWRP material under humid condition has better cooling capability when compared with their dry condition, since dried SFWRP material has lower thermal conductivity, and lack of water inside the sample also leads to low rate of moisture evaporation.

## 3. Model Establishment and Governing Equations

### 3.1. Model Hypothesis

**Figure 4:**
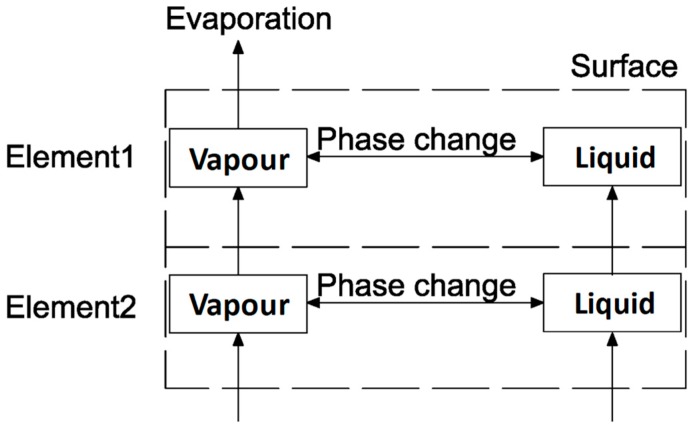
The mass transport of water moisture in SFWRP material.

The governing equations were applied to simulate the evaporation behavior of SFWRP material, in which the mass conservation equation, the momentum equation and the energy equation were taken into account to control the water saturation, fluid velocity and temperature of each point. The mass transport relationship between water vapor and water in the specimen can be seen in Figure 4, the flow rate of water vapor and air can be determined using Darcy’s law and Fick’s law of diffusion. The flow velocity of water was also determined by the Darcy’s law due to the its low flow velocity. As for the energy equation of the SFWRP material, the fluid flow term is added to the solid heat diffusion equation according to the material property and boundary condition.

**Figure 5:**
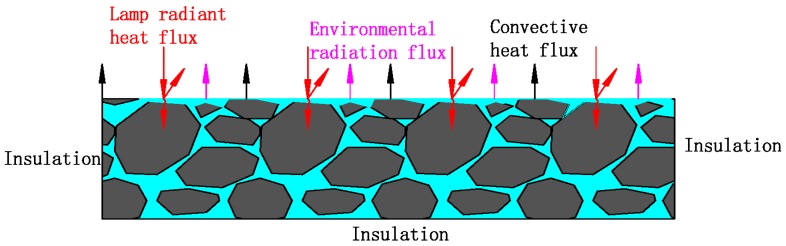
Heat exchange between environment and SFWRP material.

Figure 5 illustrates the heat exchange between environment and SFWRP material. The boundary heat flux of natural convective heat transfer was calculated with the aid of fitting parameters captured from a laboratory experiment. The boundary evaporation quantity calculation formula is established by using the similar relationship of heat and mass transfer. The model parameters of SFWRP material and asphalt mixture under waterless conditions were measured in experiment, and the thermodynamic parameters of water and air were taken from the literature [22,23].

In a laboratory test, the rutting plate sample of SFWRP material and asphalt mixture samples are manufactured according to regulation [21]; the mix ratio was designed based on the general performance of the sample [22,24]. In addition, the mechanical properties of asphalt mixture with material design were considered in the material design phase as well [25,26]. The base asphalt adopted for this study is KRY 70# Asphalt Binder, whose penetration grade is between 60 and 80; the properties of the asphalt binder used in this paper are shown in Table 1. In Table 1, the RTFO test is asphalt rolling thin film oven test which is used to simulate the short-term aging of asphalt binder.

The side and bottom of the specimen are covered with heat insulation material (thickness 5 cm) with thermal conductivity less than 0.023 W·(m·K)^−1^, which is much smaller than the thermal conductivity of the specimen. Also, the hydrophobic film material is placed around the specimen to prevent both moisture and gas phase from being connected to the environment through the bottom surface and the side surface of specimen. Therefore, the bottom surface and side surface of the sample can be considered to have no mass exchange with environment, which is consistent with the boundary condition of the model mentioned before. The upper and lower parts of the test piece are square; the sensor is placed at the center of the test piece, and the light source is also located at the central point on top of the test piece surface. The whole combination is consequently a symmetrical structure, so the test piece can be simplified as a one-dimensional geometric model for analysis in numerical simulation.

Therefore, the basic assumption of the model can be figured out as follows:

(1) Local thermodynamic equilibrium assumptions. Namely, there is no heat transfer between the gas phase, the liquid phase and the solid phase. This assumption can guarantee the same temperature distribution of the micro-body among different phases in the sample, and the chemical potential and pressure between the phases are in equilibrium.
(2) The mass flux due to temperature gradient is not considered (Soret effect) [27].
(3) The deformation of the porous skeleton (solid phase) is not considered, that is, the porosity of the SFWRP material is constant throughout the analysis.
(4) The influence of the matrix potential in the medium is much greater than the gravitational potential, so the effect of gravity is ignored.

### 3.2. Control Equations and Model Boundary Conditions

At the model establishment phase, the SFWRP material is considered as hygroscopic multiphase flow porous medium, whilst the asphalt mixture is regarded as the template model for solid heat transfer calculation (factors including internal pores and fluid flow are not taken into account). For any micro-body in the computational domain, the differential equation can be developed as follows [27].

The continuous Equations (1)–(3) are established according to the conservation of mass.

(1) $$\frac{\partial c_{v}}{\partial t}+\nabla \cdot n_{v}=\dot{m}$$

(2) $$\frac{\partial c_{a}}{\partial t}+\nabla \cdot n_{a}=0$$

(3) $$\rho_{w}\Phi \frac{\partial S_{w}}{\partial t}+\nabla \cdot n_{w}=-\dot{m}$$

The mass flux of dry air, water vapor and liquid water is calculated using Equations (4)–(6):

(4) $$n_{a}=-\rho_{a}\frac{k_{g}}{\mu_{g}}\nabla P-\frac{c_{g}^{2}}{\rho_{g}}M_{a}M_{v}D_{eff}\nabla \left(\left(P-p_{v}\right)/P\right)$$

(5) $$n_{v}=-\rho_{v}\frac{k_{g}}{\mu_{g}}\nabla P-\frac{c_{g}^{2}}{\rho_{g}}M_{a}M_{v}D_{eff}\nabla \left(p_{v}/P\right)$$

(6) $$n_{w}=-\rho_{w}\frac{k_{w}}{\mu_{w}}\nabla P_{w}$$

Since the temperatures of the phases are the same at any point, the energy conservation Equation (7) can be established by combining the solid phase, the liquid phase and the gas phase of the composition.

(7) $${\left(\rho c_{p}\right)}_{eff}\frac{\partial T}{\partial t}+{\left(\rho c_{p}\right)}_{f,eff}u_{f,eff}\cdot \nabla T=\nabla \cdot \left(\lambda_{eff}\nabla T\right)-LH\dot{m}$$

Since asphalt mixture model only consider the temperature variables, which is determined by Equation (8).

(8) $$\rho_{AC}c_{p,AC}\frac{\partial T_{AC}}{\partial t}=\nabla \cdot \left(\lambda_{AC}\nabla T_{AC}\right)$$

where: $n_{a}$ is mass flux of dry air, kg (m^2^ s)^−1^, $n_{v}$ is the mass flux of water vapor, kg (m^2^ s)^−1^, $n_{w}$ is the mass flux of water, kg (m^2^ s)^−1^, $\rho_{w}$ is the density of liquid water, kg/m^3^, $\rho_{g}$ is the density of gas phase, kg/m^3^, $\rho_{v}$ is the density of water vapor, kg/m^3^, $M_{g}$ is the molar mass of gas phase, which is synthesized by a certain ratio of dry air to water vapor, kg mol^−1^, $M_{a}$ is the molar mass of dry air, which is 0.028 kg mol^−1^, $M_{v}$ is the molar mass of water steam, which is 0.018 kg mol^−1^, $\mu_{g}$ is the viscosity of gas phase, *P* is gas phase pressure, Pa, $p_{v}$ is the gas phase pressure, Pa, $p_{w}$ is the pore water pressure, which is *P − P_c_*, P_c_ is the matrix suction, Pa,·S_W_ is the water saturation, Φ is the porosity, the gas phase concentration $c_{g}=\frac{\rho_{g}}{M_{g}}$, $\dot{m}$ is the phase change rate, which indicates that vapor is condensed into liquid water or liquid water is vaporized into water vapor in this paper, kg (m^3^ s)^−1^, LH is the latent heat of vaporization of water, J·Kg^−1^, *T* is the thermodynamic temperature of the SFWRP material, K, T_AC_ is the thermodynamic temperature of the asphalt mixture, *K*, *λ_eff_* is the equivalent thermal conductivity of SFWRP material, W·(m·K)^−1^, *λ_ac_* is the thermal conductivity of asphalt mixture, W·(m·K)^−1^, ${\left(\rho c\right)}_{eff}$ is the equivalent volumetric heat capacity of the SFWRP material, J·(m^3^ K)^−1^, $\rho_{AC}c_{p,AC}$ is the volumetric heat capacity of asphalt mixture, J·(m^3^ K)^−1^, $u_{eff}$ is the equivalent Darcy velocity of the fluid (water, dry air and water vapor) in the pore, m^2^·s^−1^, $D_{eff}$ is the equivalent diffusion coefficient of water vapor in the SFWRP material, m^2^·s^−1^, $D_{eff}=D_{va}\times \Phi \times \left(1-S_{w}\right)/\tau$, $D_{va}$ is the diffusion coefficient of water vapor in the atmosphere, m^2^·s^−1^, τ is the degree of curvature [28], which is set as 2.5 in this paper.

Therefore, the unknowns of the computational domain are *T*, $c_{v}$, $c_{a}$, *P*, $p_{v}$, $S_{w}$, $n_{a}$, $n_{v}$, $n_{w}$ (the mass flux vector degenerates into a scalar in one dimension). As it can be seen, there are 10 unknowns in $\dot{m}$, and there are seven solving equations, so it is necessary to add three Equations of state (9)–(11):

(9) $$c_{v}+c_{a}=PM_{g}/\left(RT\right)\times \left(1-S_{w}\right)\Phi$$

(10) $$c_{v}=p_{v}M_{v}/\left(RT\right)\times \left(1-S_{w}\right)\Phi$$

where the rate of phase change $\dot{m}$

(11) $$\dot{m}=K_{m}\left(c_{v,eq}c_{v}\right)$$

(12) $$c_{v,eq}=p_{sat}\times \frac{M_{g}}{RT}\times aw\times \left(1-S_{w}\right)\Phi$$

where p_sat_ is the pure water saturated water vapor pressure in the planar state, which can be calculated by Equation (13) [29]:

(13) $$p_{sat}=611.2\exp\left(17.62\times \frac{T-273.5}{T-30.03}\right)$$

Aw is the correction coefficient of capillary pressure [28]

(14) $$aw=\exp\left(\frac{-M_{v}p_{c}}{\rho_{w}RT}\right)$$

The capillary pressure p_c_ is linked to the liquid water saturation by the curve of water characteristics, as the Van Genuchten model has:

(15) $$S_{w}=\{\begin{array}{c} \frac{1}{{\left[1+{\left|\alpha_{0}H_{p}\right|}^{n_{0}}\right]}^{m_{0}}}\\ 1 \end{array}\;\;\;\;\;\begin{array}{c} H_{p}<0\\ H_{p}\geq 0 \end{array}$$

Thus, the number of equations is the same as that of unknowns and is solvable under certain initial conditions and boundary conditions.

Where *HP* is the height of water head, namely $m=\frac{-p_{c}}{\rho_{w}g}$, $\alpha_{0}$, $n_{0}$, $m_{0}$ are material parameters, g is the gravitational acceleration, which is 9.81 m·s^−2^, *K_m_* is the phase change equilibrium coefficient, s^−1^, which is used to characterize the speed of reaching phase equilibrium, the larger the value of *K_m_* indicates the faster the water vaporization phase changes to saturated water vapor; this value cannot be determined by any porous media method [30]. The value of *K_m_* should be no less than 100·Δ*t*^−1^ (the time step of the transient analysis) according to the trial calculation, otherwise the local phase equilibrium cannot be achieved. On the other hand, the calculation amount is too large or does not converge if the value of *K_m_* is too large. Therefore, the value of *K_m_* in this paper is set as 100·s^−1^, T is the temperature of evaporation water surface, *K*, *R* is the ideal gas constant, whose value is set as 8.314 J (mol K)^−1^.

Considering that there is no forced convection through the specimen, the dry air is not generated or absorbed in the test piece, so there is no need to consider the relative air flow caused by the change of the liquid water volume in the pore, and the dry air in the test piece is kept constant, *n_α_* = 0.

(16) $$\rho_{a}u_{g}-\frac{c_{g}^{2}M_{a}M_{v}}{\rho_{g}}D_{eff}\nabla \left(\frac{P-p_{v}}{P}\right)=0$$

So the convective velocity of the gas phase

(17) $$u_{g}=-\frac{\rho_{g}M_{a}M_{v}}{\rho_{a}M_{g}^{2}}D_{eff}\nabla \left(\frac{p_{v}}{P}\right)$$

Bringing Equation (17) into Equation (5) to get Equation (18) or Equation (19) as follows:

(18) $$n_{v}=-\rho_{v}\frac{\rho_{g}M_{a}M_{v}}{\rho_{a}M_{g}^{2}}D_{eff}\nabla \left(\frac{p_{v}}{P}\right)-\frac{\rho_{g}M_{a}M_{v}}{M_{g}^{2}}D_{eff}\nabla \left(\frac{p_{v}}{P}\right)$$

or

(19) $$n_{v}=-\left(1-\frac{\rho_{v}}{\rho_{a}}\right)\times \frac{\rho_{g}M_{a}M_{v}}{M_{g}^{2}}D_{eff}\nabla \left(\frac{p_{v}}{P}\right)$$

### 3.3. Model Parameters

As for the convective heat transfer on the surface of specimen, the Newton’s law of cooling was applied for the heat flux calculation [31]:

$$q_{c}=h\left(T_{s}-T_{e}\right)$$

where the convective heat transfer coefficient *h*:

(20) $$h=\frac{M_{u}\times \lambda }{L}$$

The test equation of sample surface is fitted with upper horizontal hot face [32]:

(21) $$\{\begin{array}{c} Nu=0.54{\left(GrPr\right)}^{1/4},\;\;10^{4}\leq GrPr\leq 10^{7}\\ Nu=0.15{\left(GrPr\right)}^{1/3},\;\;10^{7}\geq GrPr\leq 10^{11} \end{array}$$

where the Grashof number *Gr* = g*L*^3^αΔ*T*/*v*^2^, *λ* is the thermal conductivity of the atmosphere, W·(m·K)^−1^, Δ*T* is the temperature difference between the surface of the sample and the free atmosphere, *ΔT* = *T_S_* − *T_e_*, *T_S_* is the surface temperature of the specimen, *T_e_* is the ambient temperature, *K* and *L* are characteristic lengths, the width of the sample is 0.3 m, *h* is the convective heat transfer coefficient, W·(m^2^·K)^−1^, *Nu* is the Nusselt number, *v* is the gas kinematic viscosity, m^2^·s^−1^, α is the volume expansion coefficient, which is calculated based on the ideal gas formula:

$$\alpha =-\frac{1}{\rho }{\left(\frac{\partial \rho }{\partial T}\right)}_{p}=\frac{1}{T}$$

**Figure 6:**
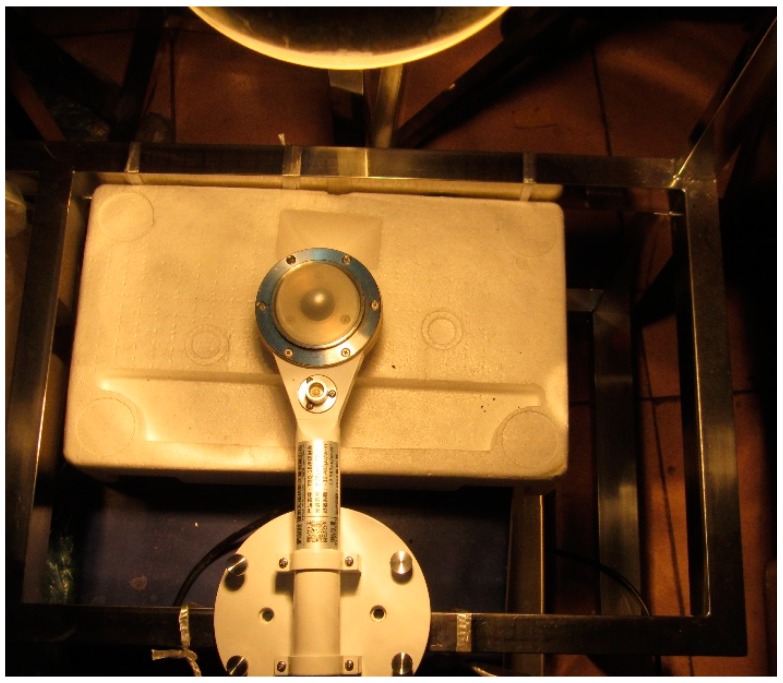
Radiant heat flux measurement.

For the radiant heat flux density on the upper surface, the reflector lamp was used as the radiant input. The surface of the test piece with real-time monitoring received a heat flux density of 954 W·m^−2^ (see Figure 6), this value is multiplied by the heat absorption rate, which is the incident radiant flux *q_r,in_*. The radiation heat transfer *q_r,out_* between the upper surface of the specimen and the surrounding environment is calculated by Stefan-Boltzmann law,

$$q_{r,out}=\varepsilon \sigma \left(T_{s}^{4}-T_{e}^{4}\right)$$

Where *ε* is the surface emissivity. The sample in this study is regarded as gray body for simplified calculation, so that the emissivity is taken as the absorption rate, and σ is the black body radiation constant, *σ* = 5.67 × 10^−8^ W·m^−2^·K^−4^.

As for water vapor flux on the surface of the specimen, namely the amount of evaporation on the surface of the specimen, this is calculated in a form similar to the Newton’s law of cooling [32]:

(22) $$E=M_{V}h_{m}\left(c_{V,S}c_{V,e}\right)\;h_{m}=\frac{Sh\times D}{L}$$

Equation (23) can be obtained from Equation (21) according to the principle of heat transfer mass transfer analogy:

(23) $$\{\begin{array}{c} Sh=0.54{\left(GrPr\right)}^{1/4},\;\;10^{4}\leq GrPr\leq 10^{7}\\ Sh=0.15{\left(GrPr\right)}^{1/3},\;\;10^{7}\leq GrPr\leq 10^{11} \end{array}\;\frac{h}{h_{m}}=\frac{Nu\times \lambda /L}{Sh\times D_{Va}/L}=\rho_{g}c_{p,g}{\left(\frac{a_{g}}{D_{Va}}\right)}^{1-n}$$

Therefore,

(24) $$h_{m}=\frac{h}{\rho_{g}c_{p,g}Le^{\left(1-n\right)}}$$

where $c_{v,e}$ is the molar concentration of water vapor in the free atmosphere, mol·m^−3^, $c_{v,s}$ is the molar concentration of water vapor on the surface of the sample, mol·m^−3^, $M_{v}$ is the molar mass of steam, which is set as 0.018 kg·mol^−1^, *ρ_g_* is the gas phase density, kg·m^−3^, *c_p,g_* is the gas phase constant pressure specific heat capacity, J/(kg·K), *a_g_* is the gas phase thermal diffusion coefficient, $a_{g}=\frac{\lambda_{g}}{\rho_{g}\times c_{p,g}}$, m^2^·s^−1^, *Le* is the Lewis number, $\mathrm{Le}=\frac{Pr}{D_{Va}}$, *Pr* is the Prandtl number, *Sh* is the Sherwood, *Sc* is the Schmidt number, $\mathrm{Sc}=\frac{v}{D_{Va}}$, *v* is the moving viscosity of the gas, m^2^·s^−1^, *n* is coefficient, *n* is set as 1/4 when $10^{4}\leq GrPr\leq 10^{7}$, and *n* is set as 1/3 when $10^{7}\leq GrPr\leq 10^{11}$. The gas parameter values representing the temperature t are taken in the calculation. The representative temperature 1/2·(*T_S_* + *T_e_*) is used as gas parameter in the calculation.

The water vaporization latent heat *LH* = 2491.146 − 2.302 × (*T* − 273.15), the liquid water saturated permeability coefficient of SFWRP tested in laboratory is 5 × 10^−8^ m·s^−1^, the Van Genuchten model was used for fitting the water characteristic curve, the parameter *α*_0_ = 0.6265, *n*_0_ = 1.6583, *m*_0_ = 1 − *n*_0_^−1^, the saturated volumetric water content of the water-retaining semi-flexible pavement is 8%, the residual water content is 0.8%, the diffusivity *D_va_* of water vapor in the air is taken according to the literature [31], the value of other parameters are shown in Table 2.

## 4. Simulation Model and Results for Validation

### 4.1. Model of Asphalt Mixture

As for the exposure test in laboratory, for both asphalt mixture and SFERP material, the procedure of the test was to heat the specimen for 5 h by indoor irradiation, then the light was turned off and cooled down for 9 h until the end of the test; during the whole test the temperature of the sample was monitored by devices mentioned before. The one-dimensional model is used to analyze the temperature field of asphalt mixture specimens under natural convection. The parameters used for model input in analysis are adopted from Table 1, and Equation (8) is used for the model governing equation, the corresponding boundary conditions are as follows:

The upper boundary (*x* = 0) is the mixed boundary of convective heat transfer and radiative heat transfer

(25) $$-\lambda_{AC}\frac{\partial T_{AC}}{\partial X}=q_{r,out}-q_{r,out}-h\left(T_{s}-T_{e}\right)$$

Where: *q_r,in_* is the light radiation absorption flux, *q_r,in_* = α_s_·954 W·m^−2^, α_s_ is the surface heat absorption rate, *q_r,out_* is the radiant flux of the test piece to the environment, $q_{r,out}=\varepsilon \sigma (T_{s}^{4}-T_{e}^{4})$, *h* is the convective heat transfer coefficient, which is calculated according to Equation (20).

The lower boundary (*x* = −0.05 m) is the adiabatic boundary $\lambda_{AC}\frac{\partial T_{AC}}{\partial X}=0$.

Initial conditions: *T_ini_* = 20.1 °C.

**Figure 7:**
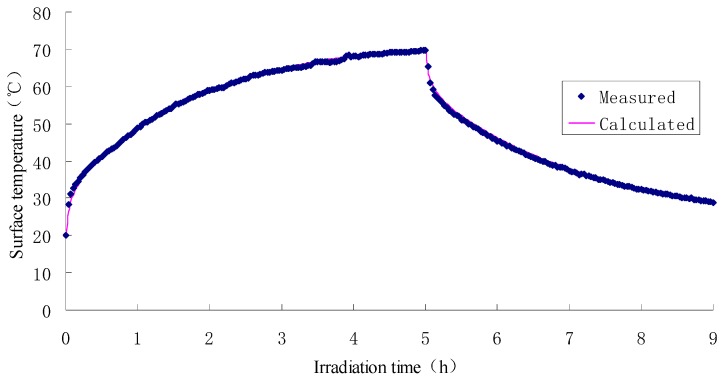
Surface temperature of asphalt concrete specimen.

The finite element modeling simulation COMSOL MULTIPHICS (Version Comsol 5.3, COMSOL Group, Stockholm, Sweden) was applied in this study to simulate the thermodynamics of the test, in which the one-dimensional transient model and solid heat transfer module were selected for simulation. The time step is set as 6 s for 100 grid units. The model is calculated by Intel Xeon(R) E3-1230v3 (E3-1230 v3, Intel, Santa Clara, CA, USA) and the main frequency is 3.30 GHz. The asphalt mixture specimen is simulated for 35 s. The comparison between the calculation results and the test results is shown in Figure 7. 

It can be seen from Figure 7 that the calculated value in FEM simulation and the measured value almost completely coincide, indicating that the natural convection heat flux calculation formula can be used for the boundary heat flux calculation under the test conditions. After 5 h of light exposure, the surface temperature of the test piece can reach at a level of 72.5 °C, which can be used for the thermal condition of the asphalt pavement.

### 4.2. Model of Semi-Flexible Water Retaining Pavement

In the laboratory, the condition of exposure test for SFWRP material is the same as the asphalt mixture, namely 5 h of indoor irradiation with 4 h of natural cooling. The one-dimensional model is used for the test of the SFWRP material and the temperature field simulation. The governing equations are (1)–(3), (6), (7), (9)–(11) and (18), The parameters used for model input in analysis are adopted from Table 1, the boundary conditions and initial conditions are as follows:

Upper boundary (*x* = 0)

*q_s_* = *q_r,in_* − *q_r,out_* − *h*(*T_S_* − *T_e_*)

*n_v_* = *h_m_*(*p_v,s_* − *p_v,e_*), *n_w_* = *0*

Where *q_s_* is the heat flux into the surface, n_v_ is the water vapor flux at the boundary, *n_w_* is the liquid water flux, *p_v,s_* is the partial water vapor partial pressure, calculated from the surface water vapor concentration, *p_v,e_* is the ambient atmospheric water The partial pressure of steam is calculated from the relative humidity. h is the convective heat transfer coefficient, calculated by Equation (20), and h_m_ is the surface mass transfer coefficient, calculated by Equation (24).

Lower boundary (*x* = −0.05 m)

*q_b_* = 0

*n_w_* = *n_v_* = 0

where the q_b_ is the heat flux into the bottom surface.

The initial conditions for indoor test simulation of water-retaining semi-flexible pavement are as follows:

In the initial condition, the temperature of the specimen was 20.1 °C, and the saturation of liquid water was 100%, the concentration of water vapor was 0, the free atmospheric temperature is 20 °C and the relative humidity is 65%.

**Figure 8:**
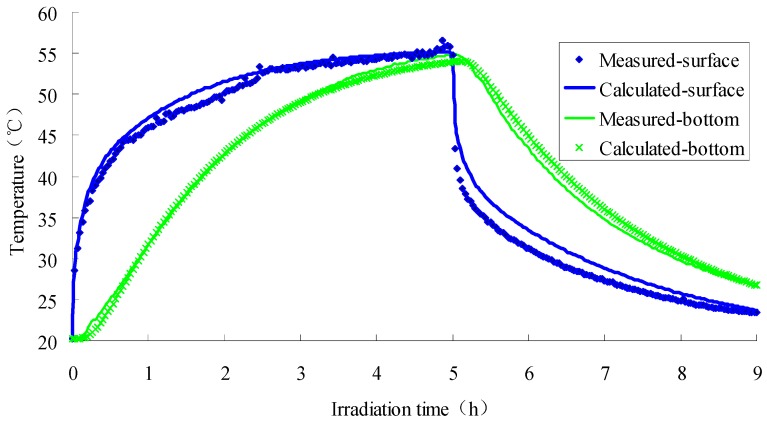
Surface and bottom temperature of water-retaining semi-flexible pavement specimen.

The finite element modeling software COMSOL MULTIPHICS was applied in this study to simulate the thermodynamics of the test, in which the one-dimensional transient model, Darcy’s law model, dilute material transport module and porous medium heat transfer module were selected for simulation. The time step is set as 6 s for 100 grid units. The model is calculated by Intel Xeon(R) E3-1230v3 and the main frequency is 3.30 GHz. The SFWRP material specimen takes 41 min for simulation. The comparison between the calculation results and the test results is shown in Figure 8. 

From Figure 8 it can be seen that the trend of the calculated value of the surface temperature of the SFWRP material interview is consistent with the trend of the measured value: a convex curve at the time of temperature rise and a concave curve at the time of temperature drop. The results captured from the comparative analysis had the basis for accurately calculating the amount of water evaporation and the temperature of the near-atmosphere. On the other hand, the calculated value of the bottom surface temperature can also be well matched with the measured value, which can guarantee to accurately simulate the temperature field of the pavement structure. Besides, there is a deviation between the simulated value and the measured value on the surface of the test piece during the period after turning off the light, indicating that the mathematical description of the rapid cooling process of the model is not accurate enough. From the figure, it can be illustrated that the calculated value is larger than the measured value. This is because during the cooling stage, the water vapor of the SFWRP material takes shorter time than it does during the temperature rising stage, so the simulated evaporation amount is smaller than measured value, which causes a higher simulated value of temperature than measured value. However, it can be seen that the maximum phase deviation of the temperature be-tween simulation and testing results is less than 3.9 °C, which indicates the potential value of for being applied in field test prediction.

**Figure 9:**
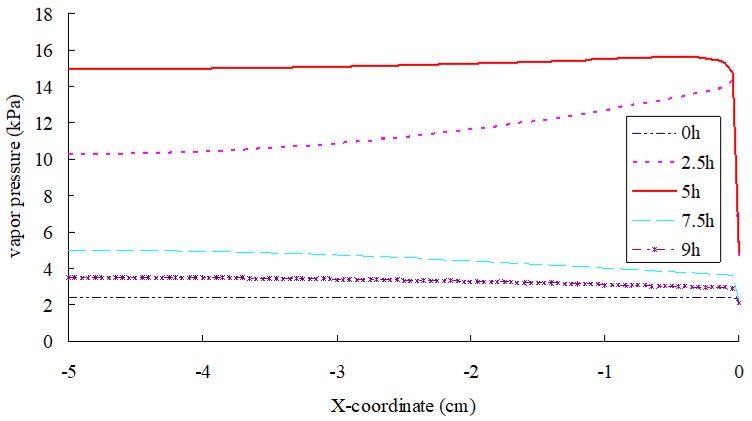
Vapor pressure distribution in SFWRP specimen (*x* axis: left-right = bottom-top).

Figure 9 illustrates the vapor distribution in SFWRP material; it can be seen that a high pressure zone of water vapor is formed during the heating process, while during the procedure of cooling a high pressure zone is formed at the bottom of specimen. Besides, from the top surface of the specimen to the depth of 2 mm inside the sample, the vapor pressure varies from large level to low. Therefore, the water vapor migrates outward due to the variation of vapor pressure. High temperature of sample surface causes large concentrations of water vapor, which leads to large volumes of water evaporation due to the variation of vapor concentration. In addition, the maximum water vapor pressure is formed at a depth of 2 mm inside the sample, which also stimulates the downward migration of water vapor below depth of 2 mm in the sample.

**Figure 10:**
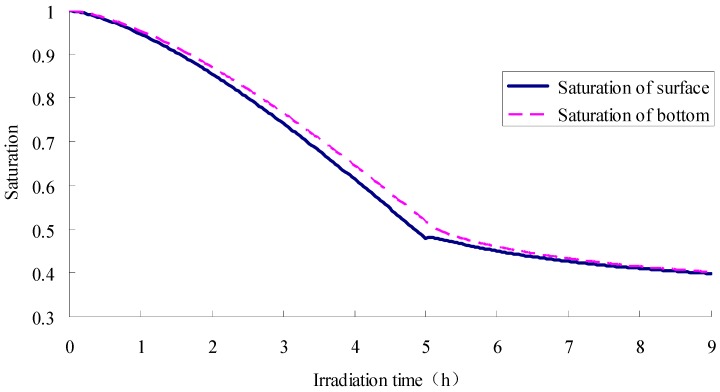
Saturation of SFWRP specimen for both top and bottom side.

**Figure 11:**
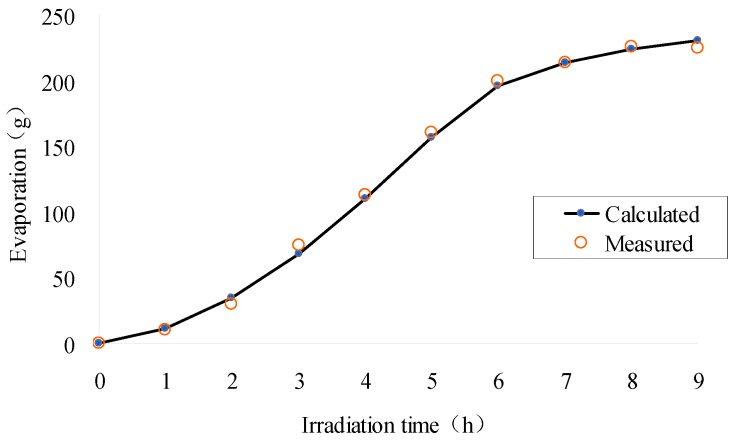
Water evaporation of SFWRP specimen during exposure time.

Figure 10 and Figure 11 describe the saturation and evaporation of SFWRP material, respectively. It can be seen that the change of trend in water saturation is opposite to which in evaporation (see Figure 10 and Figure 11). During the evaporation process, the initial evaporation is not significant, so the change rate of water saturation decrease is slow. With the increase of time, the evaporation amount becomes larger, with the decreased rate of water saturation becoming faster, and the slope of the curve becomes larger. Therefore, a convex curve is formed during heat stage, while a concave curve is formed in the cooling stage. Besides, the evaporation can still be observed after 5 h according to the figure, which means the evaporation still exists during cooling stage, but the rate of evaporation change is reduced. The decrease in evaporation will reduce the difference in saturation between the top surface and the bottom surface. When the lamp is turned off, there comes a slight increase of saturation on the top surface. From the change of water saturation at different positions in the specimen, the surface water loss is significantly faster than other places, and the surface saturation is always less than the saturation of the bottom surface, so the water always migrates from the bottom surface to the top surface. It can also be concluded from Figure 11 that the results of the simulation are consistent with the data measured in laboratory, so the liability of the model has been validated from the perspective of mass transfer.

### 4.3. Simulation of Model Template for Further Application

It is confirmed that the calculation results of the SFWRP material coupling model is basically consistent with the measured values according to the above analysis, so the model can be applied for further study of the evaporation behavior of the SFWRP material.

**Figure 12:**
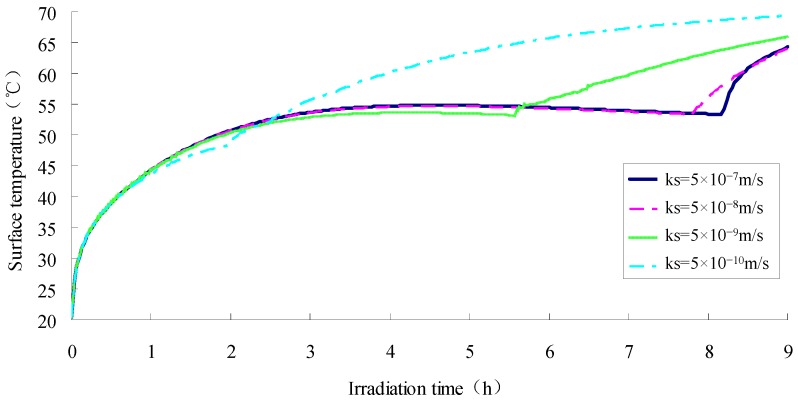
Surface temperature under different permeability coefficients.

**Figure 13:**
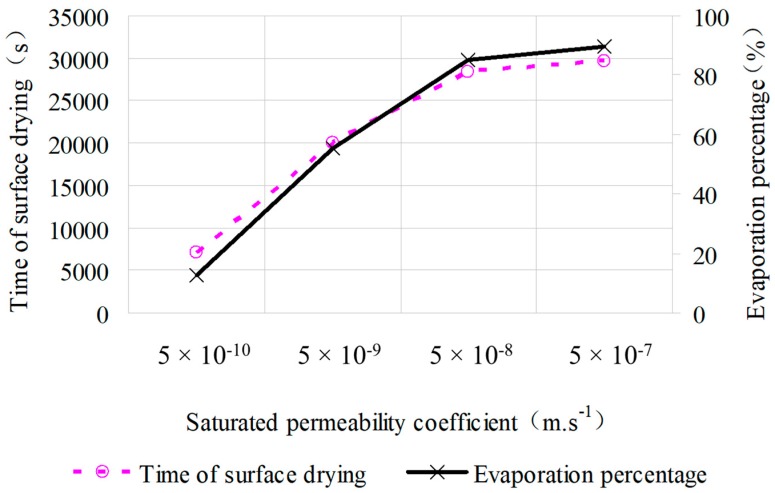
Relationship between evaporation and permeability coefficient.

In order to simulate the total amount of solar radiation during a day, the duration of the illumination heating has been extended from 5 h to 9 h, which can cause a generation of radiation in total at 3.09 × 10^7^ J. In addition, the other parameters in this model are kept as above. The surface temperature of specimen with different degrees of saturation (*ks* = 5 × 10^−7^ m·s^−1^, *ks* = 5 × 10^−8^ m·s^−1^, *ks* = 5 × 10^−9^ m·s^−1^, *ks* = 5 × 10^−10^ m·s^−1^,) were simulated in during 9 h of heat radiation; the results of simulation are shown in Figure 12, and the relationship between water evaporation and permeability of specimen are illustrated in Figure 13. As can be concluded from these two figures, all the samples with the four different permeability coefficients will finally reach the dry condition. The sample with the smaller saturated permeable coefficient will cause the faster rate of moisture evaporation, and the phase change level will move downward, so the temperature of the top surface will quickly rise during heating phase. It is known from Figure 13 that there remains 44.8% of moisture in the sample when its surface is already dry, which indicates the poor rate of water utilization. Besides, the moisture which remained in the sample is harmful to the durability of pavement structure if it cannot evaporate. Therefore, the saturated permeability coefficient of the SFWRP material should not be less than 5 × 10^−9^ m·s^−1^ according to the simulation.

**Figure 14:**
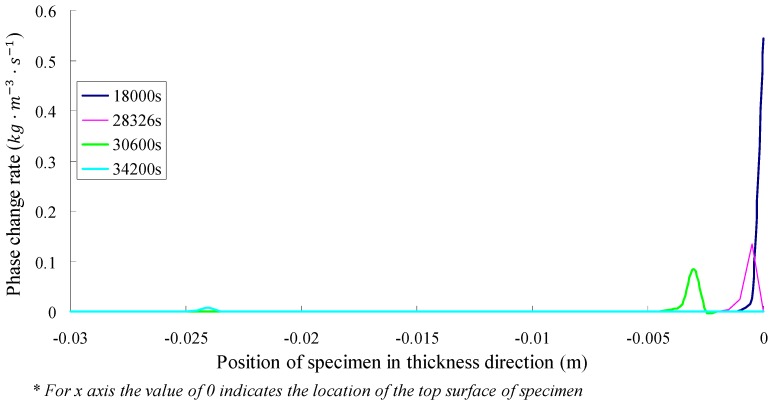
Position migration of phase change.

**Figure 15:**
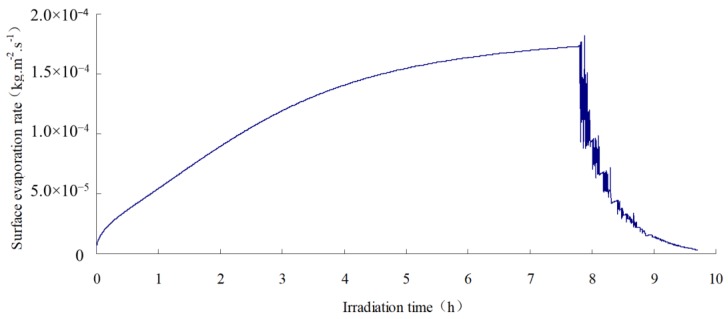
Changes of surface evaporation rate.

The SFWRP sample with saturated permeability coefficient of 5 × 10^−8^ m·s^−1^ was simulated for further study. In the simulation, the sample was subjected to the exposure reflection test until the bottom side of the sample is dry (see Figure 14 and Figure 15). According to the results of the simulation, it took 34,950 s for the exposure test to dry the bottom side of the sample, and the top surface was dry at 28,326 s, at which the range area of phase change of SFWRP material reached the maximum level; this area extended from the top surface to the depth of 3.5 mm. In the soil evaporation model, 5~20 mm is commonly used as the influence depth of surface evaporation. Therefore, the influence depth of the SFWRP material is smaller in surface evaporation when compared with solid material.

As can be seen from the figure, the phase change occurs in a tiny range which near the drier side, namely the top side. This phenomenon indicates that there is a frontal edge of evaporation in the specimen. From Figure 15, it can be seen that the evaporation volume decreases significantly when the top surface is dry due to radiation, although the surface temperature is already at a high value. Therefore, the improvement of SFWRP permeability is recommended to be taken into account from the perspective of cooling surface temperature for permeable pavement design. Since the water retention and strength of the mortar in the SFWRP material struggle to reach a balance stage, the SFWRP should adopt the mortar considering the water seepage and water retention, and the base asphalt mixture can provide sufficient strength both in cohesive bond and interlocking; the SFWRP material should be placed on the top layer in pavement structure, which can effectively motivate the migration of water vapor to the surface. 

## 5. Conclusions

In this paper, the evaporation behavior of SFWRP material was investigated by exposure reflection test in laboratory and numerical simulation. A new manufacturing and testing approach of the expo-sure test was developed in this study. The self-coding program in Finite Element Modeling (FEM) software COMSAL MULTIPHICS was used to develop the model of SFWRP material for water evaporation simulation. The main findings of this paper are as follows:

- The thermodynamic based prediction model of SFWRP considering humidity was developed; this model can be used to evaluate the evaporation behavior of SFWRP based on multi-phase thermal-mass theory. In addition, the exposure test in laboratory was also designed and con-ducted to validate this model. Noting that there is no need to measure the temperature of field pavement, which is time saving and economically beneficial as well.
- The SFWRP material under humid conditions has better cooling capability when compared with asphalt pavement, since SFWRP material has lower thermal conductivity, and the water remained inside the sample also leads to moisture evaporation. Besides, the evaluation of the cooling capability of SFWRP material is not efficient until the surface temperature of the sample is higher than 40 °C, this condition can be satisfied in field test at noon or later during the day time (for area where the sun exposure temperature is higher than 40 °C during day time).
- The saturated permeability coefficient of the SFWRP material should not be less than 5 × 10^−9^ m·s^−1^ according to the simulation.
- According to the simulation, before the top surface of SFWRP is dried, the phase change of inside material occurs in a region of 3.5 mm below the top surface, which is less than the influence depth in the soil evaporation model. On the other hand, the phase transition occurs in a small range near the drier end.
- The SFWRP should adopt the mortar considering the water seepage and water retention, and the base asphalt mixture can provide sufficient strength both in cohesive bond and interlocking; the SFWRP material should be placed on the top layer in pavement structure, which can effectively motivate the migration of water vapor to the surface.

## Figures and Tables

**Table 1:** Asphalt binder properties.

Index		Test Result	Results from RTFO Test	
Penetration (100 g 5 s)	15 °C	25.1	-	-
	25 °C	64.1	Mass Loss (%)	0.116
	30 °C	115.3	-	-
135 °C Viscosity (Pa·s)		0.709	5 °C Ductility (cm)	2.8
5 °C Ductility (cm)		7.9	-	-
Softening Point TR and B (°C)		48.7	Penetration Ratio (%)	74.6

**Table 2:** Material parameter of indoor evaporation test simulation.

Parameter	SFWRP (Dry)	Water	Water Vapor	Dry Air	Asphalt Concrete
Density *ρ*/Kg·m^−3^	2120	992.2	-	1.128	2356
Thermal Conductivity *λ*/w·(m·K)^−1^	1.010	0.635	0.0206	0.0276	1.112
Specific heat capacity *cp*/J·(kg·K)^−1^	734	4174	1885.3	1005	823
Absorption rate *α_s_*	0.85	-	-	-	0.93
Emissivity *ε*	0.85	-	-	-	0.93

Note: The thermodynamic parameters of water, steam and dry air are adopted from the literature [32], which is the value at 40 °C, and other parameters are measured in laboratory.
